# The Role of General Anesthetic Drug Selection in Cancer Outcome

**DOI:** 10.1155/2021/2563093

**Published:** 2021-10-07

**Authors:** Koichi Yuki

**Affiliations:** Department of Anesthesiology, Critical Care and Pain Medicine, Cardiac Anesthesia Division, Boston Children's Hospital, Boston, USA

## Abstract

Cancer remains to be the leading cause of death globally. Surgery is a mainstay treatment for solid tumors. Thus, it is critical to optimize perioperative care. Anesthesia is a requisite component for surgical tumor resection, and general anesthesia is given in the vast majority of tumor resection cases. Because anesthetics are growingly recognized as immunomodulators, it is critical to optimize anesthetic regimens for cancer surgery if the selection can affect outcomes. Here, we reviewed the role of volatile and intravenous anesthesia used for cancer surgery in cancer recurrence.

## 1. Introduction

Cancer remains to be the leading cause of death globally. Despite ongoing advancement in chemotherapy, radiation therapy, and immunotherapy, surgery remains to be a mainstay treatment for solid tumors [1]. However, local tumor recurrence and/or distal metastasis after surgical resection remain to be the main cause of morbidities and mortalities in solid tumors [2]. Thus, mitigating the chance of local tumor recurrence and/or distal metastases would be critical to improve the outcomes for patients.

Since the public demonstration of ether anesthesia in 1846, the importance of anesthesia in surgical procedures was widely recognized. Clearly, anesthesia has become a requisite component of surgery. Surgical resection may paradoxically create a period of vulnerability during which tumor cells disseminated in the process of manipulation of the tumor mass can surpass host immune functions for defense [3, 4]. With the appreciation of immunomodulatory properties of anesthesia, potential approaches to modulate cancer outcomes after surgical resection by anesthetics have been investigated: (1) the selection of general anesthetic drugs, and (2) the use of regional analgesia including neuraxial and paravertebral blocks, which spares the amount of general anesthesia. General anesthesia is administered in the vast majority of surgical cases for cancer resection by volatile anesthetics, intravenous anesthetics, or both. The current knowledge on the role of general anesthetic drug selection in cancer outcomes was reviewed.

## 2. Clinical Outcome Study

The landmark paper by Wigmore et al. in 2016 really ignited the discussion of whether intravenous anesthetics or volatile anesthetics should be used as general anesthetics for cancer resection surgery. They performed a retrospective, propensity-matched cohort analysis of 7,030 patients who had various types of cancer surgery and reported improved overall survival in patients given intravenous anesthetic propofol rather than volatile anesthetics (15.6% vs. 22.8% five-year mortality after surgery, respectively; *p* < 0.001) [5]. Since then, a number of investigators have retrospectively examined the outcomes after surgical resection of various cancers under total intravenous anesthesia (TIVA) vs. volatile anesthetics (Table 1). Propofol has been a major intravenous anesthetic used for this purpose. Isoflurane, sevoflurane, and desflurane are the main volatile anesthetics in current clinical use. Enflurane was used as a volatile anesthetic in the past and included in some of retrospective studies, but it is no longer available for clinical use due to its sides including nephrotoxicity.

### 2.1. Breast Cancer

Breast cancer is the most common type of malignancy in women. According to GLOBOCAN 2012, breast cancer is the leading cause of cancer-related deaths [6]. The role of TIVA vs. volatile anesthetics has been studied most in breast cancer resection surgery. Enlund et al. examined 1,837 radical breast cancer surgeries either under propofol anesthesia (620 cases) or sevoflurane anesthesia (1,217 cases) [7]. The 5-year survival was not different between the two groups. Kim et al. compared 2,533 breast cancer surgeries under volatile anesthesia (sevoflurane, desflurane, isoflurane, or enflurane) and 56 cases under TIVA [8]. There was no difference in recurrence-free survival between the two groups (*p* = 0.709). Lee et al. examined 325 modified radical mastectomies done either under propofol anesthesia (173 cases) or sevoflurane anesthesia (152 cases). The propofol group had longer recurrence-free survival (*p* = 0.037) than the sevoflurane group, but there was no difference in overall survival between the two groups (*p* = 0.383) [9]. Huang et al. examined 976 breast cancer surgical cases either under propofol anesthesia (344 cases) or desflurane anesthesia (592 cases) [10]. Following propensity match, there was no statistical difference of five-year survival rates or recurrence between the two groups (*p* = 0.454). In the study by Wigmore et al. described above, the subgroup analysis of breast cancer did not show a significant difference in the overall survival between TIVA and volatile anesthesia despite they demonstrated difference in all cancers [11]. Yoo et al. examined 5,331 patients who underwent breast cancer surgery either under TIVA (3,085 cases) or volatile anesthetics (isoflurane, sevoflurane, desflurane, and enflurane) (2,246 cases) [12]. After propensity score matching including the subtype of breast cancer, there was no significant difference in recurrence-free survival or overall survival between the two groups. Overall, all the studies except Lee's study showed no difference in postoperative cancer outcomes between TIVA and volatile anesthesia.

### 2.2. Gastrointestinal Cancers

Esophageal cancer is one of the most fatal malignancies with very poor overall five-year survival rates (10-40%) [13, 14]. Jun et al. examined 922 adult patients who underwent elective esophageal cancer either under TIVA or volatile anesthetics [15]. 191 patients received volatile anesthetics (isoflurane, sevoflurane, and desflurane), and 731 patients received TIVA. The volatile anesthetic arm was associated with worse overall survival (*p* < 0.001) and recurrence-free survival (*p* < 0.001). Even after the propensity matching, the volatile anesthetic arm was associated with worse overall survival (*p* = 0.006) and recurrence-free survival (*p* = 0.006).

Gastric cancer is the second most common cause of global cancer mortality. Zheng et al. examined 2,856 patients who underwent gastric cancer surgery either under TIVA (1,506 cases) or sevoflurane anesthesia (1,350 cases) [16]. In this study, all patients underwent laparotomy for cancer resection. The TIVA group was associated with better overall survival before and after propensity score matching (*p* < 0.001). Oh et al. examined 4,609 patients who underwent gastric cancer surgery either under TIVA (816 cases) or volatile anesthesia (sevoflurane or desflurane) (3,791 cases) [17]. Although the 1-year overall mortality was higher in the volatile anesthesia group before propensity matching (*p* = 0.012), the mortality did not differ after matching (*p* = 0.774). Different from the study by Zheng et al., more than 70% of cases were done laparoscopically.

Enlund et al. compared 695 surgical cases for colon cancer either under propofol anesthesia (179 cases) or sevoflurane anesthesia (516 cases) [7]. The propofol group had better 1-year and 5-year survival (*p* < 0.001 and *p* < 0.05, respectively). Wu et al. examined 706 patients who underwent colon surgery either under propofol anesthesia (657 cases) or desflurane anesthesia (706 cases) [18]. After propensity matching, the propofol anesthesia group had a better survival, irrespective of lower tumor-node-metastasis stage (*p* < 0.001), higher tumor-node-metastasis stage (*p* < 0.001), presence of metastases (*p* = 0.002), or absence of metastases (*p* = 0.016).

Enlund et al. also compared rectal cancer surgery. 104 cases and 202 cases were performed under propofol anesthesia or sevoflurane anesthesia, respectively. There was no difference in 1-year and 5-year survival between the groups. In addition to the low number of patients compared in this study, however, half of the patients in this study were for reoperative surgery, which may add the complexity to this cohort to draw the conclusion about the effect of anesthetic drugs on postoperative outcome.

Overall, the majority of studies examining gastrointestinal cancer surgery demonstrated that TIVA offered a better recurrence-free survival and overall survival. Furthermore, in the study by Wigmore et al., the subgroup of patients requiring gastrointestinal surgery had better survival under TIVA, in line with other studies [11].

### 2.3. Hepatobiliary System Cancer

Lai et al. examined 944 patients who underwent hepatectomy for hepatocellular carcinoma under propofol anesthesia (452 cases) or desflurane anesthesia (492 cases) [19]. In the propensity-matched analysis, the propofol anesthesia group had a better survival (*p* < 0.001). Koo et al. examined 259 patients who underwent laparoscopic hepatic surgery under propofol anesthesia (121 cases) or volatile anesthesia (138 cases) [20]. Propofol anesthesia was associated with a significantly decreased 2-year recurrence survival (*p* = 0.029). Of note, volatile anesthetics used in this study were not specified in this study. Lai et al. examined 70 patients who underwent open intrahepatic cholangiocarcinoma surgery under propofol anesthesia (34 cases) or desflurane anesthesia (36 cases) [21]. After propensity matching, propofol anesthesia was associated with a better survival (*p* = 0.032).

### 2.4. Lung Cancer

Lung cancer is one of the leading causes of cancer-related death worldwide. Non-small-cell lung cancer is the most common type of lung cancer, accounting for 80% of lung cancers. Oh et al. examined 943 cases of non-small-cell lung cancer resection either under TIVA (749 cases) or sevoflurane anesthetics (194 cases). There was no significant difference in overall survival and recurrence-free survival between the two arms before and after propensity matching [22].

Despite an inherent limitation that the studies described above are all retrospective in nature, they demonstrated that TIVA could provide better recurrence-free outcome and overall survival in gastrointestinal cancer and hepatobiliary cancer surgery. In addition to the type of cancer surgery, the invasiveness of surgery may be also an important contributor, as demonstrated that TIVA improved survival of post gastric cancer surgery done by laparotomy, while it did not affect the outcome of patients largely done via laparoscopy. Breast cancer surgery is likely less invasive than surgeries such as abdominal surgeries, for example. The fact that breast cancer surgery was seldomly affected by the type of general anesthetic drugs could support the hypothesis that the invasiveness of surgery plays a role in cancer recurrence. The idea that surgical invasiveness is associated with cancer recurrence is supported by an animal study; in murine orthotopic models of spontaneous postoperative metastasis, simple primary breast tumor resection did not progress to metastatic diseases unless accompanied by the surgical stress and tissue injury of a laparotomy [23]. Certainly, a prospective randomized control study is critical to clarify the role of general anesthetic drugs in postsurgical outcome of cancer patients. Another question is if there is any difference in isoflurane, sevoflurane, or desflurane-based volatile anesthesia, although isoflurane, sevoflurane, and desflurane are all called halogenated ethers, because they are derivatives of volatile anesthetic ether [24]. Despite they are structurally similar, a growing literature suggests that they have some difference in target molecules [25–27], indicating that the selection among currently available volatile anesthetics may be also an important consideration. For example, surgical site infection was significantly higher in sevoflurane-based general anesthesia than in desflurane-based general anesthesia [28].

## 3. Potential Mechanism of Anesthesia-Mediated Tumor Recurrence

### 3.1. Mechanism of Cancer Recurrence

Postoperative cancer recurrence frequently takes the form of metastatic diseases [29]. Tumor cell dissemination to and colonization at distant sites is considered to occur during surgery [1]. Circulating tumor cells (CTCs) are detectable in the majority of patients with solid tumors [30]. The presence of a high number of CTCs is associated with a poor tumor prognosis [31]. The elevation in the number of CTCs has been described after cancer resection surgery [32–34]. However, the presence of CTCs is not necessarily equal to metastasis. Host immune cells usually survey CTCs for eradication. For CTCs to colonize, they need to escape from host immune cells such as NK cells and reach to “premetastatic niche.” Surgical wound, infection site, or traumatized tissue is an attracted site for CTCs to be colonized, serving as a premetastatic niche, because these places have supporting extracellular matrix compared to the vascular system, inflammatory, and prothrombotic responses to repair traumatized tissues providing a significant milieu of mediators beneficial for tumor survival. In addition, surgical stress can activate the sympathomedullary (SAM) axis to secrete catecholamines. Catecolamines orchestrate immune suppression by mobilizing leukocytes out of the circulation as well as reducing their effector functions via the activation of adrenergic receptor-mediated signaling within leukocytes, which includes diminished numbers and cytolytic activity of NK cells and increased levels of Th2 cells [35, 36]. Thus, general anesthetic drugs that minimize surgical stress by affecting SAM axis to limit immunosuppression and directly mitigate tumor viability and growth but directly augment host immune cells would be ideal ones to provide general anesthesia.

### 3.2. The Effect of Anesthetics on Tumor Cells and Host Immune Cells

#### 3.2.1. Tumor Viability and Growth

Hypoxia-inducible factors (HIFs) are a family of transcription factors that regulate a vast array of genes involved in critical aspects of tumor activities such as cell proliferation, angiogenesis, glucose metabolism, and cell invasion [37]. Thus, the effect of anesthetics on the HIF pathway has been a major interest. Isoflurane upregulated the levels of HIF-1*α* and HIF-2*α* via phosphoinositide 3-kinase (PI3K)/Akt/mechanistic target of the rapamycin (mTOR) pathway and enhanced human renal cancer cell RCC4 cell migration and proliferation [38]. Similarly, isoflurane induced HIF-1*α* expression in prostate cancer cells PC3 with an increase in proliferation and migration [39]. Isoflurane increased VEGF, angiopoietin 1, matrix metallopeptidase- (MMP-) 2, and MMP-9 expression, which is the downstream event of the HIF-1 signaling pathway, compatible with increased angiogenesis and invasion [40]. Similar to isoflurane, sevoflurane accelerated proliferation of cervical cancer cells and breast cancer cells [41, 42]. Desflurane also enhanced migration [43]. However, the effect of volatile anesthetics on tumor cells can be cell-type dependent. For example, isoflurane exposure significantly increased caspase-3 activation and reduced cell viability of H4 human neuroglioma cells [44]. Hepatocellular carcinoma cell (HCC) viability was also attenuated by isoflurane [45]. Sevoflurane inhibited the proliferation of neck squamous cell cancer (HNSCC) and lung adenocarcinoma [46, 47]. Desflurane attenuated the proliferation of colorectal cancer cells [48]. Because the HIF signaling pathway is ubiquitously important, it is not clear how to explain the difference in phenotypes.

Then, how about propofol? Propofol exposure for 24 hours reduced the levels of HIF-1*α* and attenuated the invasion and migration of breast cancer cells MDA-MB-231 cells [49]. In line, propofol attenuated HIF-1*α* expression in PC3 cells. However, propofol exposure for much shorter durations (1, 4, and 12 hours) rather increased proliferation and migration of MDA-MB-231 cells in a dose- and time-dependent manner [50].

Taken together, the effect of general anesthetic drugs on tumor cells may be dependent on a number of factors including the type of tumor cells and exposure duration. Mechanistic investigation needs to be determined so that future modification of anesthetics can be considered.

#### 3.2.2. Host Immune Cellular Function

Among various types of leukocytes critical for cancer immunology, natural killer (NK) cells and T cells are two predominant cytotoxic effector cells that are the major components of cell-mediated immune responses.

#### 3.2.3. NK Cell Function

NK cells are a phenotypically distinct population of lymphocytes (CD56+/CD3-) that lyse tumor cells using constitutively expressed lytic machinery independent of prior immunization. NK cells survey and conjugate with tumor cells devoid of major histocompatibility complex (MHC) class I and polarize lytic granules toward them. Subsequent degranulation of lytic proteins such as perforin, granzyme, and Fas ligands leads tumor cells to apoptosis. The correlation of perioperative NK cell suppression with tumor recurrence and mortality after surgical resection of colorectal and lung cancer suggests that adequate, perioperative NK cell function is critical to minimize postresection cancer recurrence [51, 52].

The number of NK cells in the perioperative period was studied by Bartal et al. [53]. At postoperative 12 hours, NK cell number was reduced after major surgeries, while it was similar compared to the baseline after minor surgeries. Because catecholamines produced as a result of SAM axis activation in surgery regulate circulating leukocyte numbers, this is predictable. The effect of propofol and desflurane-based anesthesia on NK cell number was examined in patients undergoing breast cancer surgery [54]. NK cells were reduced at 24 hours in both groups, but there was no difference between the two anesthetic groups. Liu et al. examined NK cell counts in patients who underwent radical hysterectomy either under propofol anesthesia or sevoflurane anesthesia [55]. Postoperative NK cell number was significantly less in both groups, but the degree of reduction was more profound in the sevoflurane group. It is unclear if the difference between the two studies was due to difference between the two volatile anesthetics sevoflurane and desflurane or due to difference in surgical procedures. Because the apoptosis of NK cells did not differ between propofol and sevoflurane anesthesia [56], it is possible that sevoflurane arm had less attenuation of catecholamine production/stress responses than propofol arm, leading to the mobilization of NK cells from the peripheral blood, and desflurane might have attenuated stress responses similar to propofol. Studies comparing stress responses including the levels of catecholamines under different general anesthetic drugs can clarify this question.

Then, general anesthetic drugs affect NK cell function differently? NK cells express a number of activating and inhibitor receptors, which would contribute to their effector functions. One of activating receptors is leukocyte function-associated antigen-1 (LFA-1). The binding of LFA-1 to intercellular adhesion molecule-1 (ICAM-1) on tumor cells induces lytic granule polarization and NK-cell-mediated cytolysis [57, 58]. At clinically relevant concentrations, volatile anesthetics isoflurane and sevoflurane act as LFA-1 allosteric inhibitors [59–62]. Propofol, on the other hand, did not show significant inhibition of LFA-1 at the clinically relevant concentrations [59, 63, 64]. In line with these findings, isoflurane and sevoflurane significantly attenuated NK cell-mediated tumor cytotoxicity [65]. In contrast, intravenous agents propofol, etomidate, ketamine, midazolam, fentanyl, and dexmedetomidine did not attenuate NK cell-mediated tumor cytotoxicity at clinically relevant concentrations. No effect on tumor cytotoxicity by propofol was also shown in the study by Melamed et al. where MADB106 cells were intravenously injected into rats [66]. So far, the effect of desflurane on LFA-1 and NK cell cytotoxicity has not been known.

Type I interferons activate NK cells for tumor cytotoxicity [67]. Isoflurane exposure attenuated interferon-induced NK cell activity *in vivo* [68]. It is not known about the role of sevoflurane, desflurane, or propofol in type I interferon-mediated NK cell activation so far.

A number of studies examined the direct effect of anesthetics on NK cell functions, but Buckley et al. examined the effect of serum under different anesthetics on NK cell functions [69]. They coincubated serum from patients who underwent breast cancer surgery under propofol and paravertebral block anesthesia or sevoflurane-based anesthesia with healthy NK cells. NK cells coincubated with the serum from patients receiving propofol-based anesthesia exhibited a strong cytotoxicity, while NK cells coincubated with the serum from sevoflurane-based anesthesia showed a marked impairment of cytotoxicity. The serum from the sevoflurane group showed less interleukin- (IL-) 1*β* and IL-10 levels. The serum level of type I interferons was not measured in this study. IL-1*β* is a costimulatory of NK cells [70]. Thus, it is possible that a reduction in IL-1*β* in the sevoflurane group may be in part responsible for their results. Because the propofol group uses regional block, however, the difference in serum composition between the two anesthetic groups may not necessarily be attributed solely to the difference in general anesthetic drugs.

Overall, a number of studies have supported that volatile anesthetics attenuated the function of NK cells compared to propofol-based anesthesia, but further studies are needed to clarify if all the halogenated ether derivatives act similarly. ICAM-1 is often expressed on a group of tumor cells, but some have its very limited expression. The studies that tested the effect of anesthetics on NK cell cytotoxicity used K562 cells and YAC-1 cells, both of which express ICAM-1 highly. Whether or not volatile anesthetics affect NK cell-mediated tumor cytotoxicity when tumor cells have very limited ICAM-1 expression remains to be determined. Whether or not transcriptomic pattern of tumors affect the effect of general anesthetic drugs on host immune responses such as NK cells would be an important question to be investigated in the future.

#### 3.2.4. T Cells

Comparing the effects of volatile anesthetics vs. intravenous anesthetic on T cells has been performed on a very limited basis. Liu et al. examined T cell counts in patients who underwent radical hysterectomy either under propofol anesthesia or sevoflurane anesthesia [55]. In both groups, postoperative T cell numbers were significantly less than preoperative values. Postoperative T cell counts were significantly lower in the sevoflurane group than in the propofol group. This may be explained by a potential difference in the effect of sevoflurane and propofol on stress response, as described in NK cell section. Future studies are needed to clarify this matter.

Helper T (Th) cells play an important role in tumor immunity. Th1 effector cells produce IL-2 and interferon-*γ*, tumor necrosis factor (TNF)-*α*, which primarily mediate antitumor immunity. Th2 effector cells produce IL-4, IL-5, IL-6, IL-10, and IL-13 but can promote cancer progression. Propofol did not affect the Th1/Th2 ratio, while isoflurane significantly decreased Th1/Th2 [71]. Sevoflurane attenuated the Th1/Th2 ratio [72]. In contrast, desflurane preserved the Th1/Th2 balance [54]. The clinical importance of Th1/Th2 ratio modulation by different anesthetic drugs needs to be determined in the future.

## 4. Conclusion

A number of retrospective clinical studies showed that propofol-based TIVA might offer outcome benefit in cancer patients compared to volatile anesthetics in a subset of cancer surgeries. The effect of volatile anesthetics on tumor cells and immune cells may be in part supportive of these clinical studies. However, prospective randomized control trials are needed to clarify. Furthermore, whether or not there is any difference among different volatile anesthetics needs to be investigated.

## Figures and Tables

**Table 1:** Outcome of cancer surgery and anesthesia.

Type of study	Cancer type	Outcome	Power	Reference
Retrospective	Various cancers	5-year mortality VA 22.8% vs. TIVA 15.6%	*p* < 0.001	Wigmore et al. [5]
Retrospective	Breast cancer	5-year mortality no difference between sevoflurane and propofol arms	n.s.	Enlund et al. [7]
Retrospective	Breast cancer	No difference in recurrence-free survival between VA and TIVA	*p* = 0.646	Kim et al. [8]
Retrospective	Breast cancer	Propofol arm had longer recurrence-free survival than sevoflurane arm; no difference in overall survival	*p* = 0.037 (recurrence free); *p* = 0.383 (survival)	Lee et al. [9]
Retrospective	Breast cancer	Propofol arm had longer recurrence-free survival than sevoflurane arm; no difference in overall survival	*p* = 0.454	Huang et al. [10]
Retrospective	Breast cancer	No difference in recurrence-free survival between VA and TIVA	*p* = 0.782	Yoo et al. [12]
Retrospective	Esophageal cancer	VA was associated with worse overall survival and recurrence-free survival	*p* < 0.001 (overall survival, recurrence-free survival)	Jun et al. [15]
Retrospective	Gastric cancer	TIVA was associated with better overall survival than sevoflurane	*p* < 0.001	Zheng et al. [16]
Retrospective	Gastric cancer	VA had higher 1-year mortality than TIVA	*p* = 0.012	Oh et al. [17]
Retrospective	Colon cancer	Propofol had better 1-year and 5-year survival than sevoflurane	*p* < 0.001 (1-year survival); *p* < 0.05 (5-year survival)	Enlund et al. [7]
Retrospective	Colon cancer	Propofol had better survival than desflurane	*p* < 0.001-*p* = 0.016	Wu et al. [18]
Retrospective	Rectal cancer	No difference in 1-year and 5-year survival between propofol and sevoflurane	n.s.	Enlund et al. [7]
Retrospective	Hepatocellular cancer	Propofol had better survival than desflurane	*p* < 0.001	Lai et al. [19]
Retrospective	Hepatocellular cancer	Propofol was associated with less 2-year recurrence survival than VA	*p* = 0.029	Koo et al. [20]
Retrospective	Cholangiocarcinoma	Propofol was associated with better survival than desflurane	*p* = 0.032	Lai et al. [21]
Retrospective	Non-small-lung cancer	No difference in overall survival and recurrence-free survival between TIVA and sevoflurane	*p* = 0.072 (overall survival); *p* = 0.862 (recurrence-free survival)	Oh et al. [22]

## Data Availability

All the data are available in the manuscript.

## References

[1] Hiller J. G., Perry N. J., Poulogiannis G., Riedel B., Sloan E. K. (2018). Perioperative events influence cancer recurrence risk after surgery. *Nature Reviews. Clinical Oncology*.

[2] Gottschalk A., Sharma S., Ford J., Durieux M. E., Tiouririne M. (2010). Review article: the role of the perioperative period in recurrence after cancer surgery. *Anesthesia and Analgesia*.

[3] Tavare A. N., Perry N. J., Benzonana L. L., Takata M., Ma D. (2012). Cancer recurrence after surgery: direct and indirect effects of anesthetic agents∗. *International Journal of Cancer*.

[4] Demicheli R., Retsky M. W., Hrushesky W. J., Baum M., Gukas I. D. (2008). The effects of surgery on tumor growth: a century of investigations. *Annals of Oncology*.

[5] Wigmore T. J., Mohammed K., Jhanji S. (2016). Long-term survival for patients undergoing volatile versus IV anesthesia for cancer surgery: a retrospective analysis. *Anesthesiology*.

[6] Ferlay J., Soerjomataram I., Dikshit R. (2015). Cancer incidence and mortality worldwide: sources, methods and major patterns in GLOBOCAN 2012. *International Journal of Cancer*.

[7] Enlund M., Berglund A., Andreasson K., Cicek C., Enlund A., Bergkvist L. (2014). The choice of anaesthetic--sevoflurane or propofol--and outcome from cancer surgery: a retrospective analysis. *Upsala Journal of Medical Sciences*.

[8] Kim M. H., Kim D. W., Kim J. H., Lee K. Y., Park S., Yoo Y. C. (2017). Does the type of anesthesia really affect the recurrence-free survival after breast cancer surgery?. *Oncotarget*.

[9] Lee J. H., Kang S. H., Kim Y., Kim H. A., Kim B. S. (2016). Effects of propofol-based total intravenous anesthesia on recurrence and overall survival in patients after modified radical mastectomy: a retrospective study. *Korean Journal of Anesthesiology*.

[10] Huang Y. H., Lee M. S., Lou Y. S. (2019). Propofol-based total intravenous anesthesia did not improve survival compared to desflurane anesthesia in breast cancer surgery. *PLoS One*.

[11] Sessler D. I., Riedel B. (2019). Anesthesia and cancer Recurrence. *Anesthesiology*.

[12] Yoo S., Lee H.-B., Han W. (2019). Total intravenous AnesthesiaversusInhalation anesthesia for breast cancer Surgery. *Anesthesiology*.

[13] D'Amico T. A. (2007). Outcomes after surgery for esophageal cancer. *Gastrointestinal cancer research*.

[14] Huang F. L., Yu S. J. (2018). Esophageal cancer: risk factors, genetic association, and treatment. *Asian Journal of Surgery*.

[15] Jun I. J., Jo J. Y., Kim J. I. (2017). Impact of anesthetic agents on overall and recurrence-free survival in patients undergoing esophageal cancer surgery: a retrospective observational study. *Scientific Reports*.

[16] Zheng X., Wang Y., Dong L. (2018). Effects of propofol-based total intravenous anesthesia on gastric cancer: a retrospective study. *OncoTargets and therapy*.

[17] Oh T. K., Kim H. H., Jeon Y. T. (2019). Retrospective analysis of 1-year mortality after gastric cancer surgery: total intravenous anesthesia versus volatile anesthesia. *Acta Anaesthesiologica Scandinavica*.

[18] Wu Z. F., Lee M. S., Wong C. S. (2018). Propofol-based total intravenous anesthesia is associated with better survival than desflurane anesthesia in colon cancer surgery. *Anesthesiology*.

[19] Lai H. C., Lee M. S., Lin C. (2019). Propofol-based total intravenous anaesthesia is associated with better survival than desflurane anaesthesia in hepatectomy for hepatocellular carcinoma: a retrospective cohort study. *British Journal of Anaesthesia*.

[20] Koo B. W., Lim D. J., Oh A. Y., Na H. S. (2020). Retrospective comparison between the effects of propofol and inhalation anesthetics on postoperative recurrence of early- and intermediate-stage hepatocellular carcinoma. *Medical Principles and Practice*.

[21] Lai H. C., Lee M. S., Lin K. T. (2019). Propofol-based total intravenous anesthesia is associated with better survival than desflurane anesthesia in intrahepatic cholangiocarcinoma surgery. *Medicine*.

[22] Oh T. K., Kim K., Jheon S. (2018). Long-term oncologic outcomes for patients undergoing volatile versus intravenous anesthesia for non-small cell lung cancer Surgery. *Cancer Control*.

[23] Glasner A., Avraham R., Rosenne E. (2010). Improving survival rates in two models of spontaneous postoperative metastasis in mice by combined administration of a beta-adrenergic antagonist and a cyclooxygenase-2 inhibitor. *Journal of Immunology*.

[24] Yuki K., Hou L., Shibamura-Fujiogi M., Koutsogiannaki S., Soriano S. G. (2021). Mechanistic consideration of the effect of perioperative volatile anesthetics on phagocytes. *Clinical Immunology*.

[25] Jung S., Yuki K. (2016). Differential effects of volatile anesthetics on leukocyte integrin macrophage-1 antigen. *Journal of Immunotoxicology*.

[26] Zha H., Matsunami E., Blazon-Brown N. (2019). Volatile anesthetics affect macrophage phagocytosis. *PLoS One*.

[27] Mitsui Y., Hou L., Huang X., Odegard K. C., Pereira L. M., Yuki K. (2020). Volatile anesthetic sevoflurane attenuates toll-like receptor 1/2 activation. *Anesthesia and analgesia*.

[28] Yamamoto S., Nagamine Y., Miyashita T. (2020). Perioperative and anesthetic risk factors of surgical site infection in patients undergoing pancreaticoduodenectomy: a retrospective cohort study. *PLoS One*.

[29] Demicheli R., Retsky M. W., Hrushesky W. J., Baum M. (2007). Tumor dormancy and surgery-driven interruption of dormancy in breast cancer: learning from failures. *Nature Clinical Practice. Oncology*.

[30] Nagrath S., Sequist L. V., Maheswaran S. (2007). Isolation of rare circulating tumour cells in cancer patients by microchip technology. *Nature*.

[31] Rahbari N. N., Aigner M., Thorlund K. (2010). Meta-analysis shows that detection of circulating tumor cells indicates poor prognosis in patients with colorectal cancer. *Gastroenterology*.

[32] Brown D. C., Purushotham A. D., Birnie G. D., George W. D. (1995). Detection of intraoperative tumor cell dissemination in patients with breast cancer by use of reverse transcription and polymerase chain reaction. *Surgery*.

[33] Hashimoto M., Tanaka F., Yoneda K. (2014). Significant increase in circulating tumour cells in pulmonary venous blood during surgical manipulation in patients with primary lung cancer. *Interactive Cardiovascular and Thoracic Surgery*.

[34] Peach G., Kim C., Zacharakis E., Purkayastha S., Ziprin P. (2010). Prognostic significance of circulating tumour cells following surgical resection of colorectal cancers: a systematic review. *British Journal of Cancer*.

[35] Scanzano A., Cosentino M. (2015). Adrenergic regulation of innate immunity: a review. *Frontiers in Pharmacology*.

[36] Ince L. M., Weber J., Scheiermann C. (2019). Control of leukocyte trafficking by stress-associated hormones. *Frontiers in Immunology*.

[37] Semenza G., Targeting L. (2003). Targeting HIF-1 for cancer therapy. *Nature Reviews Cancer*.

[38] Benzonana L. L., Perry N. J. S., Watts H. R. (2013). Isoflurane, a commonly used volatile anesthetic, enhances renal cancer growth and malignant potential via the hypoxia-inducible factor cellular signaling pathway in vitro. *Anesthesiology*.

[39] Huang H., Benzonana L. L., Zhao H. (2014). Prostate cancer cell malignancy via modulation of HIF-1 *α* pathway with isoflurane and propofol alone and in combination. *British Journal of Cancer*.

[40] Luo X., Zhao H., Hennah L. (2015). Impact of isoflurane on malignant capability of ovarian cancer _in vitro_^‡^. *British Journal of Anaesthesia*.

[41] Xue F., Xu Y., Song Y., Zhang W., Li R., Zhu X. (2019). The effects of sevoflurane on the progression and cisplatinum sensitivity of cervical cancer Cells. *Drug design, development and therapy*.

[42] Ecimovic P., McHugh B., Murray D., Doran P., Buggy D. J. (2013). Effects of sevoflurane on breast cancer cell function in vitro. *Anticancer Research*.

[43] Iwasaki M., Zhao H., Jaffer T. (2016). Volatile anaesthetics enhance the metastasis related cellular signalling including CXCR2 of ovarian cancer cells. *Oncotarget*.

[44] Xie Z., Dong Y., Maeda U. (2006). The common inhalation anesthetic isoflurane induces apoptosis and increases amyloid beta protein levels. *Anesthesiology*.

[45] Hu J., Hu J., Jiao H., Li Q. (2018). Anesthetic effects of isoflurane and the molecular mechanism underlying isoflurane‑inhibited aggressiveness of hepatic carcinoma. *Molecular Medicine Reports*.

[46] Yang Y., Hu R., Yan J. (2017). Sevoflurane inhibits the malignant potential of head and neck squamous cell carcinoma via activating the hypoxia‑inducible factor-1*α* signaling pathway in vitro. *International Journal of Molecular Medicine*.

[47] Liang H., Gu M. N., Yang C. X., Wang H. B., Wen X. J., Zhou Q. L. (2011). Sevoflurane inhibits proliferation, induces apoptosis, and blocks cell cycle progression of lung carcinoma cells. *Asian Pacific Journal of Cancer Prevention*.

[48] Muller-Edenborn B., Roth-Z'graggen B., Bartnicka K. (2012). Volatile anesthetics reduce invasion of colorectal cancer cells through down-regulation of matrix metalloproteinase-9. *Anesthesiology*.

[49] Li Q., Zhang L., Han Y., Jiang Z., Wang Q. (2012). Propofol reduces MMPs expression by inhibiting NF-*κ*B activity in human MDA- MB-231 cells. *Biomedicine & Pharmacotherapy*.

[50] Meng C., Song L., Wang J., Li D., Liu Y., Cui X. (2017). Propofol induces proliferation partially via downregulation of p53 protein and promotes migration via activation of the Nrf2 pathway in human breast cancer cell line MDA-MB-231. *Oncology Reports*.

[51] Tartter P. I., Steinberg B., Barron D. M., Martinelli G. (1987). The prognostic significance of natural killer cytotoxicity in patients with colorectal cancer. *Archives of Surgery*.

[52] Fujisawa T., Yamaguchi Y. (1997). Autologous tumor killing activity as a prognostic factor in primary resected nonsmall cell carcinoma of the lung. *Cancer*.

[53] Bartal I., Melamed R., Greenfeld K. (2010). Immune perturbations in patients along the perioperative period: alterations in cell surface markers and leukocyte subtypes before and after surgery. *Brain, Behavior, and Immunity*.

[54] Woo J. H., Baik H. J., Kim C. H. (2015). Effect of propofol and desflurane on immune cell populations in breast cancer patients: a randomized trial. *Journal of Korean Medical Science*.

[55] Liu S., Gu X., Zhu L. (2016). Effects of propofol and sevoflurane on perioperative immune response in patients undergoing laparoscopic radical hysterectomy for cervical cancer. *Medicine*.

[56] Oh C. S., Lee J., Yoon T. G. (2018). Effect of equipotent doses of PropofolversusSevoflurane anesthesia on regulatory T cells after breast cancer surgery. *Anesthesiology*.

[57] Weitz-Schmidt G., Chreng S., Riek S. (2009). Allosteric LFA-1 inhibitors modulate natural killer cell function. *Molecular Pharmacology*.

[58] Kohl S., Springer T. A., Schmalstieg F. C., Loo L. S., Anderson D. C. (1984). Defective natural killer cytotoxicity and polymorphonuclear leukocyte antibody-dependent cellular cytotoxicity in patients with LFA-1/OKM-1 deficiency. *Journal of Immunology*.

[59] Koutsogiannaki S., Schaefers M. M., Okuno T. (2017). From the cover: prolonged exposure to volatile anesthetic isoflurane worsens the outcome of polymicrobial abdominal sepsis. *Toxicological Sciences*.

[60] Yuki K., Astrof N. S., Bracken C., Soriano S. G., Shimaoka M. (2010). Sevoflurane binds and allosterically blocks integrin lymphocyte function-associated antigen-1. *Anesthesiology*.

[61] Yuki K., Astrof N. S., Bracken C. (2008). The volatile anesthetic isoflurane perturbs conformational activation of integrin LFA-1 by binding to the allosteric regulatory cavity. *The FASEB Journal*.

[62] Yuki K., Bu W., Xi J., Sen M., Shimaoka M., Eckenhoff R. G. (2012). Isoflurane binds and stabilizes a closed conformation of the leukocyte function-associated antigen-1. *The FASEB Journal*.

[63] Yuki K., Bu W., Xi J., Shimaoka M., Eckenhoff R. (2013). Propofol shares the binding site with isoflurane and sevoflurane on leukocyte function-associated antigen-1. *Anesthesia and Analgesia*.

[64] Yuki K., Soriano S. G., Shimaoka M. (2011). Sedative drug modulates T-cell and lymphocyte function-associated antigen-1 function. *Anesthesia and Analgesia*.

[65] Tazawa K., Koutsogiannaki S., Chamberlain M., Yuki K. (2017). The effect of different anesthetics on tumor cytotoxicity by natural killer cells. *Toxicology Letters*.

[66] Melamed R., Bar-Yosef S., Shakhar G., Shakhar K., Ben-Eliyahu S. (2003). Suppression of natural killer cell activity and promotion of tumor metastasis by ketamine, thiopental, and halothane, but not by propofol: mediating mechanisms and prophylactic measures. *Anesthesia and Analgesia*.

[67] Paolini R., Bernardini G., Molfetta R., Santoni A. (2015). NK cells and interferons. *Cytokine & Growth Factor Reviews*.

[68] Markovic S. N., Knight P. R., Murasko D. M. (1993). Inhibition of interferon stimulation of natural killer cell activity in mice anesthetized with halothane or isoflurane. *Anesthesiology*.

[69] Buckley A., McQuaid S., Johnson P., Buggy D. J. (2014). Effect of anaesthetic technique on the natural killer cell anti-tumour activity of serum from women undergoing breast cancer surgery: a pilot study. *British Journal of Anaesthesia*.

[70] Cooper M. A., Fehniger T. . A., Ponnappan A., Mehta V., Wewers M. . D., Caligiuri M. . A. (2001). Interleukin-1*β* costimulates interferon-*γ* production by human natural killer cells. *European Journal of Immunology*.

[71] Inada T., Yamanouchi Y., Jomura S. (2004). Effect of propofol and isoflurane anaesthesia on the immune response to surgery. *Anaesthesia*.

[72] Wada H., Seki S., Takahashi T. (2007). Combined spinal and general anesthesia attenuates liver metastasis by preserving TH1/TH2 cytokine balance. *Anesthesiology*.

